# Overcoming the challenge of persistent left atrial appendage thrombosis: a case report of successful left atrial appendage occlusion using a cerebral protection system

**DOI:** 10.1093/ehjcr/ytag138

**Published:** 2026-03-04

**Authors:** Darko Althoff, Hannes Alessandrini, Jan-Per Wenzel, Roland Richard Tilz

**Affiliations:** Department of Rhythmology, University Heart Center Lübeck, University Hospital Schleswig-Holstein, Ratzeburger Allee 160, 23538 Lübeck, Germany; Department of Rhythmology, University Heart Center Lübeck, University Hospital Schleswig-Holstein, Ratzeburger Allee 160, 23538 Lübeck, Germany; Department of Rhythmology, University Heart Center Lübeck, University Hospital Schleswig-Holstein, Ratzeburger Allee 160, 23538 Lübeck, Germany; Department of Rhythmology, University Heart Center Lübeck, University Hospital Schleswig-Holstein, Ratzeburger Allee 160, 23538 Lübeck, Germany

**Keywords:** Atrial fibrillation, Cerebral embolism, Left atrial appendage closure, Stroke prevention, Case report

## Abstract

**Background:**

Atrial fibrillation significantly increases the risk of ischaemic stroke, primarily due to left atrial appendage (LAA) thrombosis. Percutaneous left atrial appendage occlusion (LAAO) offers an alternative approach to oral anticoagulation. However, the presence of LAA thrombosis has been considered a contraindication due to the risk of cerebral embolization.

**Case summary:**

An 82-year-old woman presented to our department with dyspnoea and history of non-valvular persistent atrial fibrillation as well as heart failure. Serial transoesophageal echocardiography revealed persistent LAA thrombosis despite optimal oral anticoagulation. As a therapeutic alternative, percutaneous LAA closure with cerebral embolic protection was carried out successfully with no severe adverse events reported. In the following, the patient also underwent cryoballoon pulmonary vein isolation to achieve rhythm control. In the follow-up examination, the anticoagulation was replaced with aspirin and transthoracic echocardiography showed normalization of the left ventricular ejection fraction in sinus rhythm.

**Discussion:**

This case report illustrates that in highly selected patients with persistent LAA thrombus despite prolonged, optimized anticoagulation, LAAO performed under meticulous procedural precautions and cerebral embolic protection may represent a carefully justified, individualized bail-out strategy

Learning points

- In some patients with non-valvular atrial fibrillation, persistent left atrial appendage (LAA) thrombosis can occur despite optimal oral anticoagulation.
- Left atrial appendage occlusion (LAAO) combined with a cerebral protection system (CPS) offers a safe and feasible treatment strategy with present LAA thrombus.

## Introduction

The risk of ischaemic stroke is increased five-fold in patients with atrial fibrillation (AF). Left atrial appendage (LAA) thrombosis has been identified as the main cause of ischaemic stroke in these patients. Oral anticoagulation (OAC) remains the cornerstone of thromboembolic risk mitigation in AF, effectively reducing the incidence of LAA thrombus formation and subsequent ischaemic events. However, anticoagulation therapy is inherently associated with an elevated risk of bleeding complications.^1,2^ Despite use of direct oral anticoagulants (DOACs) or vitamin K antagonists (VKAs), ischaemic stroke can occur in some patients.^3^ A meta-analysis reported an LAA thrombus prevalence of 2.7% among patients with AF or atrial flutter receiving optimal anticoagulation with no significant difference between those treated with DOAC and those receiving VKA.^4^

Percutaneous left atrial appendage occlusion (LAAO) has emerged as a device-based alternative for stroke prevention in patients with AF and an elevated thromboembolic risk. The WATCHMAN FLX device (Boston Scientific, Minneapolis, Minnesota) showed a high incidence of anatomic closure and low rates of periprocedural adverse events.^1,5^ However, in the presence of LAA thrombosis, LAAO procedures carry a significantly heightened risk of thromboembolism, particularly cerebral embolization, necessitating additional precautions. Cerebral embolic protection devices, initially developed for transcatheter aortic valve implantation (TAVI), have been adapted for LAAO to mitigate this risk. The SENTINEL cerebral protection system (CPS) (Boston Scientific, Minneapolis, Minnesota) has been proven both safe and feasible in LAAO procedures with LAA thrombosis being present.^6,7^ In this case report, an 82-year-old woman with AF and persistent LAA thrombus underwent percutaneous LAAO using the WATCHMAN FLX as well as the SENTINEL CPS.

## Summary figure

**Figure:**
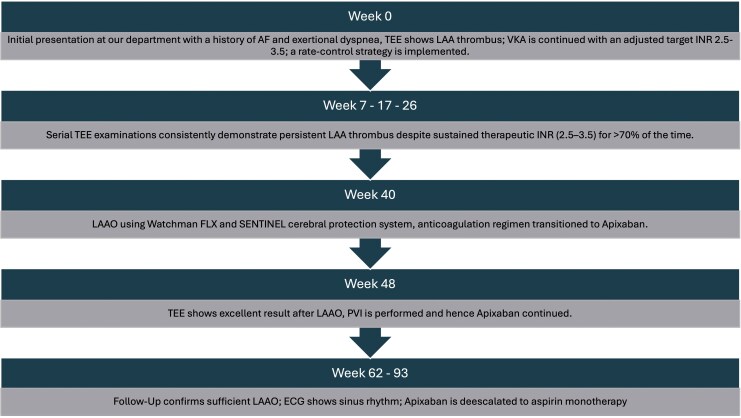


## Case presentation

**Figure 1:**
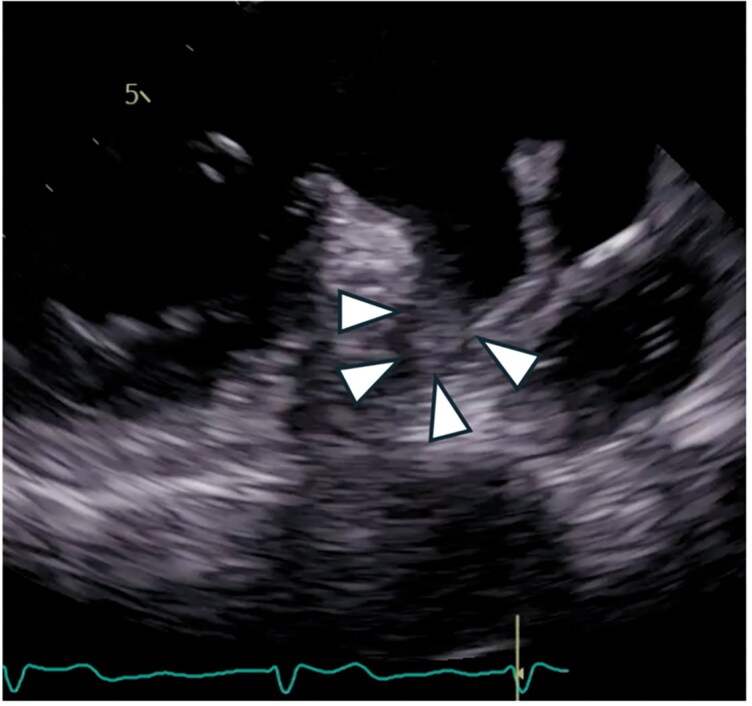
Pre-procedural transoesophageal echocardiography depicting left atrial appendage thrombus (triangles).

An 82-year-old woman was initially referred to our department with history of non-valvular, persistent AF (CHA2DS2-VA score = 4, HAS-BLED score = 3), heart failure indeterminate aetiology with mildly reduced ejection fraction (left ventricular ejection fraction = 40%) and rheumatoid arthritis. The physical examination showed no signs of cardiac decompensation. Coronary angiography excluded significant coronary artery disease; the transthoracic echocardiography confirmed a left ventricular ejection fraction of 40% with no further signs of structural cardiomyopathy. Given these findings, tachycardia-induced cardiomyopathy was assumed as the most likely cause of reduced ejection fraction. Oral anticoagulation with phenprocoumon (target international normalized ratio (INR) 2–3) had been established. The INR upon presentation was in the therapeutic range, though there were ambulatory determinations with suboptimal INR < 2. Therefore, transoesophageal echocardiography (TEE) was performed before attempting rhythm control. Transoesophageal echocardiography revealed LAA thrombosis; hence, a target INR of 2.5–3.5 and rate control was adopted (*Figure 1*).

Despite adequate anticoagulation (INR persistently within the upper therapeutic range), serial TEE examinations at 7, 17, and 26 weeks continued to demonstrate a well-organized LAA thrombus. Given the lack of thrombus resolution despite OAC, percutaneous LAA closure with cerebral embolic protection was deemed the most appropriate therapeutic alternative.

**Figure 2:**
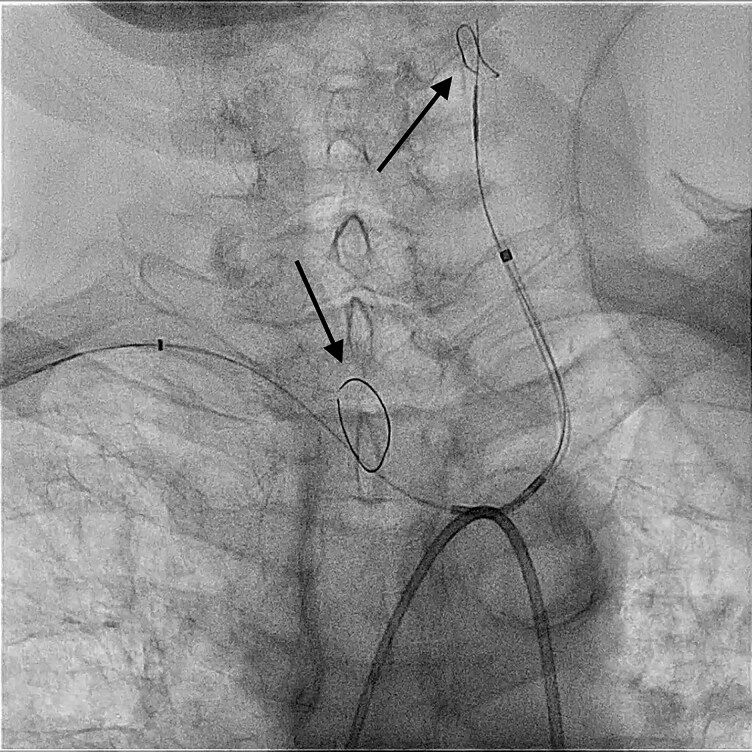
Fluoroscopic image showing the positioning of the SENTINEL cerebral protection system. The proximal filter has been placed at the junction of the brachiocephalic trunk and the distal filter in the left common carotid artery (arrows).

Written consent was obtained, and the patient was scheduled for the procedure. The procedure was performed under deep sedation and TEE guidance. An 8 French (F) sheath was introduced into the right femoral artery using ultrasound. Right radial artery access was obtained via a 6 F sheath. Two ultrasound-guided venous punctures were performed in the right femoral vein (8 and 14 F WATCHMAN TruSeal Sheath), and a multipolar catheter (Biosense Webster, Diamond Bar, CA, USA) was positioned in the coronary sinus. Angiography of the aortic arch, brachiocephalic trunk, and left common carotid artery was conducted using a pigtail catheter inserted into the ascending aorta via the femoral artery sheath to assess the anatomy. The SENTINEL system was deployed through the radial artery access into the target vessels, and the proximal filter was placed into the brachiocephalic trunk and the distal filter in the left common carotid artery (*Figure 2*).

**Figure 3:**
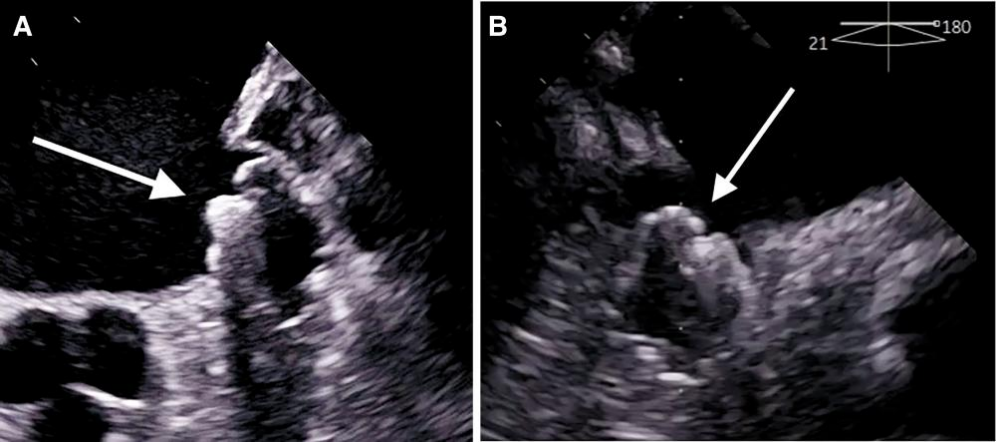
Transoesophageal echocardiography depicting left atrial appendage closure after implantation of the WATCHMAN FLX device (arrows).

**Figure 4:**
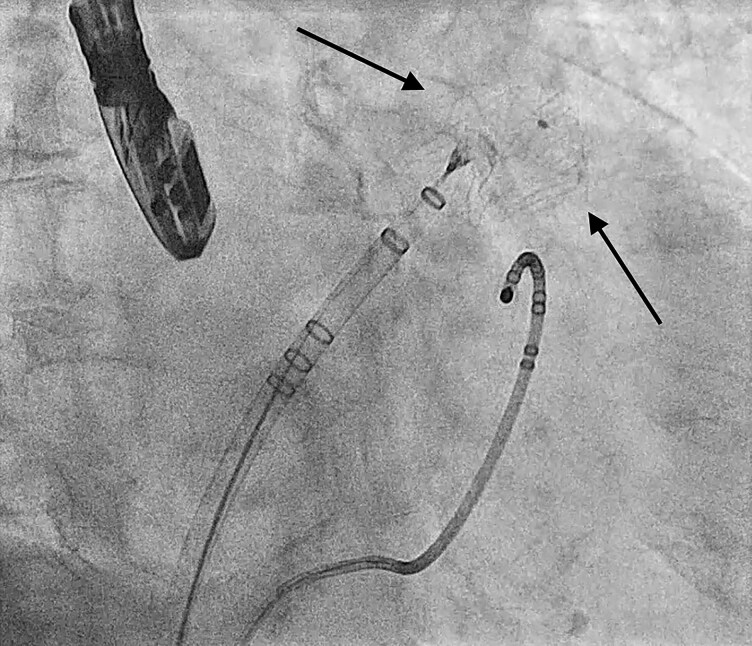
Fluoroscopic image during implantation of the WATCHMAN FLX device (arrows).

Left atrial access was achieved using a single transseptal approach and an 8.5 F transseptal sheath (SL1 sheath, St. Jude Medical, Inc., St. Paul, MN, USA) via the right femoral vein under fluoroscopic guidance. The SL1 sheath was then exchanged for the WATCHMAN TruSeal sheath over a guidewire. No selective angiography of the LAA was performed to reduce the risk of thrombus dislodgement. The landing zone diameter was measured using TEE. The appropriate device was loaded onto the delivery system, advanced into the LA through the WATCHMAN TruSeal sheath, and carefully deployed into the LAA (*Figures 3* and *Figure 4*). Proper device positioning was ensured through two- and three-dimensional multiplanar TEE. No peri-device leaks or residual gaps were detected, and stability was confirmed using a standard tug test.

Following successful device implantation, the femoral access site was secured using a vascular closure device (Angio-Seal, Terumo, Japan) for the arterial access and a figure-of-eight suture and pressure bandage for the venous access. Radial artery haemostasis was achieved using a TR-Band (Terumo, Japan) following the removal of the SENTINEL system. No periprocedural complications, including systemic embolization or ischaemic stroke, were observed. Oral anticoagulation with apixaban 5 mg b.i.d. was initiated.

Transoesophageal echocardiography performed 2 months after implantation confirmed proper device placement without any signs of leakage. Cryoballoon pulmonary vein isolation (PVI) was carried out to achieve rhythm control. During follow-up examination 3 months later, OAC was replaced with aspirin, and sinus rhythm was observed. Additionally, transthoracic echocardiography (TTE) showed normalization of the left ventricular ejection fraction in sinus rhythm.

## Discussion

In patients with AF and persisting LAA thrombus under DOAC therapy, there are several alternative anticoagulation strategies that can be considered, including low-molecular-weight heparin and high-dose VKA. However, no protocol has proven superior in thrombus dissolvement.^2,3^ The management chosen in this case diverges from current European Society of Cardiology (ESC) guidelines, which generally regard LAA thrombus as a contraindication to percutaneous LAA occlusion and recommend optimization of anticoagulation until complete thrombus resolution is documented.^1^ In contrast, existing evidence from small series and case reports suggests that LAAO with cerebral protection may be a feasible ‘bail-out’ option in selected patients with refractory LAA thrombus.^8,9^ In our patient, thrombus size remained unchanged under high-dose VKA after several follow-ups, which prompted an individualized, off-label decision to perform LAAO under cerebral protection. Upon reaching an INR ≤ 2 following the LAAO, we reverted the OAC back to apixaban to simplify management and improve patient adherence.

Other studies showed macroscopic debris in the CPS filters in about one-third of patients upon completion of the procedure, raising concerns about potential ischaemic events in vascular territories not covered by the CPS or when a CPS is not used altogether.^7,10^ Regarding our clinical case, there were no macroscopic visible clots in the filters upon removal. Neither microscopic analysis of the filters nor magnetic resonance imaging (MRI) was performed, since the procedures were not relevant for clinical management of our patient.

In conclusion, it must be explicitly acknowledged that this approach is contrary to current ESC guideline recommendations, which consider the presence of LAA thrombus a contraindication to percutaneous LAA occlusion.^1^ However, this case illustrates that in highly selected patients with persistent LAA thrombus despite prolonged, optimized anticoagulation, LAAO performed under meticulous procedural precautions and cerebral protection may represent a carefully justified, individualized bail-out strategy rather than a generalizable treatment option.

## Lead author biography


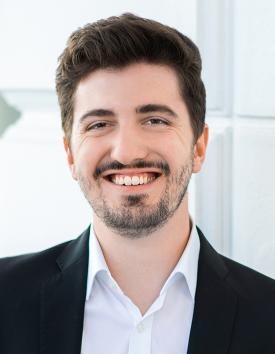


Darko Althoff is a fifth-year resident at the Department of Rhythmology, University Hospital Schleswig-Holstein. He is originally from Austria, where he graduated from the Medical University of Graz in 2020.

 

**Consent:** The authors confirm that written consent for submission and publication of this case report, including the images and associated text, has been obtained from the patient in line with the COPE guidelines.

## Data Availability

All available data are presented within the manuscript.
